# Ewing sarcoma of the head and neck region in pediatric patients: A retrospective monoinstitutional series

**DOI:** 10.1177/03008916261432403

**Published:** 2026-05-20

**Authors:** Valentina Di Ruscio, Ida Russo, Angela Di Giannatale, Evelina Miele, Elisa Meldolesi, Sabina Vennarini, Alessandra Stracuzzi, Rita Alaggio, Giada Del Baldo, Mario Zama, Urbano Urbani, Giuseppe Maria Milano

**Affiliations:** 1 Department of Oncohematology, Gene and Cell Therapy, Bambino Gesù Children's Hospital IRCCS, Rome, Italy; 2 Department of Radiation Oncology, Fondazione Policlinico Universitario A. Gemelli, IRCCS, Rome, Italy; 3 Radiotherapy Department, Fondazione IRCCS Istituto Nazionale Dei Tumori, Milan, Italy; 4 Department of Pathology, Bambino Gesù Children's Hospital IRCCS, Rome, Italy; 5 Plastic and Maxillo-Facial Surgery, Bambino Gesù Children's Hospital, IRCCS, Rome, Italy

**Keywords:** Ewing sarcoma, maxillofacial, skull, head and neck, pediatric oncology, surgery

## Abstract

Ewing sarcoma (ES) occurs in the head and neck (HN) region in fewer than 10% of cases, presenting distinct therapeutic challenges.

We retrospectively reviewed all ES cases diagnosed at our institution from 2000 to 2022.

Of 131 patients, 10 (7.6%) had primary HN tumors (median age: 10 years). Tumor locations included the paranasal sinuses (n=6), jaw (n=2), and skull (n=2). Three patients presented with metastatic disease.

All patients received first-line chemotherapy, except one who underwent upfront surgery. Local treatment included radiotherapy (RT) alone (n=4), surgery plus RT (n=4), and surgery alone (n=2). Resection was feasible in six cases, with five patients achieving R0 margins following delayed surgery after Busulfan-Melphalan (BuMel) consolidation. Reconstruction used autologous bone grafts (n=3) or free fibular flaps (n=2), with one graft failure.

One patient had local recurrence at 24 months; three with initial metastases had distant relapse at 8, 29, and 44 months. Median event-free survival (EFS) was 27 months (range: 8–44), and median overall survival (OS) was 63 months (range: 12–159). At last follow-up, five patients were in complete remission, one was receiving third-line therapy, one was lost to follow-up, and three died in disease progression.

In conclusion, metastatic disease at diagnosis remains the strongest predictor of poor outcome. Nevertheless, our findings highlight the importance of a multidisciplinary team approach, with close collaboration among radiation oncologists and maxillofacial surgeons. Such integrated teamwork may improve prognosis and help reduce long-term sequelae.

## Introduction

Ewing sarcoma (ES) is the second most common primary malignant bone cancer among children and adolescents, with a cumulative incidence of about 2.93 cases per 1,000,000 patients. These tumors most commonly involved ribs, pelvis, or long bones’ diaphysis and about 10–20% occur in soft tissues. Less than 10% arise in the head and neck region (HN), mainly in patients under 18 years of age. The most commonly affected bones are the skull, the mandible and the maxilla; more rarely paranasal sinuses.^1^

Through collaborative efforts and sequential clinical trials, ES treatment has largely evolved over the past four decades. Effective treatment requires a multimodal approach, integrating local control through surgery and/or conventional radiotherapy (RT) or proton beam radiotherapy (PT), alongside multi-agent chemotherapy (CT). Over the years, chemotherapeutic regimens, when feasible, have been reduced, in combination with advances in local control.^2^ However, in this peculiar localization, the degree of local control is often limited by the risk of collateral damage to adjacent structures and the potential short and long-term local sequelae, including functional and aesthetic sequelae, growth abnormalities with face asymmetry, endocrine disorders, and neurological or psychosocial impairment.^3^

The limited number of published case series on HN ES results in heterogeneous treatment approaches and makes it difficult to define an optimal therapeutic algorithm.

Here we report our single-center experience of HN ES among pediatric patients over a period of 20 years, with the intent of increasing knowledge and reporting our experience on multimodal management of this peculiar localization.

All patients' parents provided informed consent for participation in this retrospective study.

This study has been approved by our Institutional review board and by the Institutional Ethics Committee.

## Cases description

Between 2000 and 2022, 131 patients were diagnosed with ES at our Institution; 10 cases (7.6%) involved the head and neck (HN) region.

We retrospectively reviewed clinical records, imaging studies, and surgical, pathological, and radiotherapy reports, including data on systemic and local treatments. All histological diagnoses were confirmed by molecular analyses for EWSR1 fusion, using real-time transcription-polymerase chain reaction (RT-PCR) or Fluorescence In Situ Hybridization (FISH), and, in recent years, next-generation sequencing (NGS) with Archer FusionPlex custom panels.

Patients’ characteristics are detailed in Table 1.

**Table 1.** Patients’ characteristics.

Patients	Sex	Age at diagnosis	Clinical presentation	Tumor size	Location	Metastasis at diagnosis	Site of metastasis
1	M	13	Endocranial hypertension, vomiting and cervical pain	8 cm x 5.5 cm x 4 cm	Skull (with extension to orbit and basicranium)	N	
2	F	10	Painless swelling	3 cm x 3 cm x 5.5 cm	Jaw	Y	Lung
3	F	10	Fronto-maxillary headache, sinusitis	7 cm x 5.5 cm x 5.5 cm	sphenoid and ethmoid sinus	N	
4	M	10	Painless swelling	NA	maxillary sinus	N	
5	F	5	Painless swelling	2.8 cm x 3.8 cm x 3.2 cm	Jaw	Y	Bones, lung
6	M	12	Painless swelling	3 cm x 3.7 cm x 4 cm	maxillary sinus	Y	Bones, lung
7	M	9	Painless swelling	5.3 cm x 2.8 cm x 3.5 cm	maxillary sinus	N	
8	F	18	Painless swelling	4.2 cm x 3.5 cm x 3.9 cm	maxillary sinus	N	
9	F	3	Painless swelling, palpebral edema and right eye ptosis	3.6 cm x 3.8 cm x 3.5 cm	maxillary sinus	N	
10	M	8	Painless swelling	3.2 cm x 3 cm x 2.8 cm	skull (with extension to orbit and leptomeningeal enhancement)	N	

Legend: M: male; F: female; Y: yes; N: no.

Median age at diagnosis was 9.8 years (range 3–18), with a male-to-female ratio of 1:1.

Primary sites included the paranasal sinuses (6/10; five maxillary, one sphenoid), jaw (2/10), and skull (2/10). Three patients presented with metastatic disease (one with lung metastases; two with combined bone and lung metastases). Tumor size ranged from 3 to 5 cm in eight cases and reached up to 8 cm in two cases.

The most common presenting symptom was painless swelling (9/10), in one case associated with headache and sinusitis. One patient with skull ES presented with signs of intracranial hypertension.

Nine patients underwent incisional or fine-needle biopsy; one had primary radical resection before referral to our institution.

Treatment followed a multimodal strategy based on national and international guidelines, including CT, high-dose-CT with busulfan–melphalan (BuMel) and autologous stem cell rescue, RT or PT, and surgery. Details are described in **Table 2.**

**Table 2.** First and second-line treatments.

Patients	First-line CT treatment	Timing of surgery	Necrosis	RT	Relapse/progression	EFS (m)	Second-line treatments	OS (m)	Status at last f-up
1	EW2	Not performed	NA	Y				56	A
2	EW2	Not performed	NA	Y (T+ TLI)	Y, lung	43.6	CT, RT	69	A
3	EW2	Not performed	NA	Y				149	A
4	EW1	POST BuMEL	>90%	N				15	lost to f-up
5	EW2	POST BuMEL	>90%	Y (TLI)	Y, lung	29.4	CT, RT	64	D
6	ICE/CAV	POST BuMel	>90%	Y (T+ TLI)	Y, lung	8.9	CT, RT	12	D
7	ICE/CAV	POST BuMEL	>90%	Y	Y, local	24.9	CT, RT	33	D
8	EW1	POST BuMEL	<90%	N				108	A
9	EW1	Not performed	NA	Y				26	A
10	EW2	At diagnosis	NA	Y				53	A

Legend: CT: chemotherapy; ICE: ifosfamide-carboplatin and etoposide cycle; CAV: cyclophosphamyde-actynomicin D and vincristine; EW1: neo-adjuvant chemotherapy according to National Protocol AIEOP EW1; EW2: according to National Protocol AIEOP EW2 Protocol); NA: not applicable; T: primitive tumor; TLI: Total lung irradiation; RT: radiotherapy; EFS: event-free survival; OS: overall survival; D: dead; A: alive.

After neoadjuvant CT, radiological response was complete response (CR) in 4/10 patients (including one after primary surgery) and partial response (PR) in 6/10.

High-dose CT were performed in all patients. Four patients subsequently underwent RT alone (two photons, two protons) and three patients received RT after surgical approach. One patient, receiving surgery at onset, underwent PT before BuMel regimen. Total lung irradiation up to 12 Gy was performed in patients with lung metastasis at diagnosis. Patients received a median radiotherapy dose ranging from 41.4 to 59.4 Gy to the primary lesion.

Surgery was feasible in 6/10 patients, either at diagnosis (1/6) or after high-dose CT (5/6). All patients achieved gross total resection with wide margins. Reconstruction aimed to preserve function and aesthetics: three patients received autologous bone grafts, and two underwent fibular free flap reconstruction following hemimaxillectomy or partial mandibular resection. One patient required secondary reconstruction due to bone exposure; no further surgical complications occurred. Although necrosis could not be considered a prognostic factor in our cohort, histological evaluation showed >90% chemotherapy-induced necrosis (Picci grade 3) in four patients and <90% (grade 2) in one.

Regarding long-term toxicity, one patient with temporal bone ES developed extensive hemispheric radionecrosis seven months after RT and was treated with bevacizumab. He later developed radiation-induced corneal necrosis requiring enucleation. Three patients treated with PT experienced only mild radiodermatitis, which was resolved with topical therapy.

Local relapse occurred only in one patient, a localized ES of the maxillary sinus, at 24 months from diagnosis, while multiple lung metastatic recurrence was detected in three patients –all metastatic at onset-, at 8, 29 and 44 months since diagnosis respectively, with a median event-free survival (EFS) of 26 months (range 8-44 months). Two patients achieved a second CR, and had a second relapse at 17 and 31 months after first relapse, with a single pleural-based lesion and a diffuse bone marrow involvement, respectively.

At the time of writing, five patients in first CR are alive, one patient was treated with second-line CT and RT and is currently under third-line CT, while one patient was lost to follow-up in first CR at 15 months since diagnosis. Three patients died due to disease progression (respectively at 12, 33 and 64 months from primary diagnosis). Considering all evaluable patients, two-years EFS was 77%, while 2-years and 5-years OS were 88% and 66%, respectively.

## Conclusion

Head and neck ES are an extremely rare localization among pediatric bone sarcomas, and consequently there are few data available to guide oncological management. Despite the challenges associated with local therapy in the HN region, limited data suggests that the prognosis for this site is favorable and comparable to that of ES arising in the extremities.^4^ Several case series reported that mandibular site localization is a favorable prognostic factor, while cervical vertebrae and flat skull bones involvement are linked to a poorer prognosis.^5^ In our series two patients had mandibular disease with lung and bone metastasis at diagnosis.

It is well-known that ES is usually a chemo and radiosensitive tumor, thus leading to multimodal treatment strategies diversified over the years, including CT, RT/PT and surgery.^1^

Concerning HN patients, nowadays there are 14 large case series available, including the adult population, and the studies range from 1972 to 2015, with a significant diversification of treatment strategies, as shown in Table 3.

**Table 3.** Case series of HN ES of adult and pediatric population.

Series	Years	N. of pediatric patients (total cohort)	Median f-up (y)	OS (%)	EFS (%)	Prognostic impact of HN localization (other prognostic factors reported)	HD-CT	Timing of local control (weeks)	Type of local control	M+ (%)
Razek et al^6^	1972-1986	NR (12)	1	100	100	Favorable	NR	After CT	RT	NR
Allam et al^4^	1975-1996	NR (24)	3.4	53	30	Favorable (better OS for good responder and for jaw localization)	No	Majority of cases after neo-adjuvant CT	58% RT; 21% surgery followed by CT + RT	46
Siegal et al^7^	1972-1986	29 (32)	4.7	80	NR	Favorable (better OS for jaw localization)	No	NR	100% RT; 48% surgery + RT	NR
Desai et al^8^	1985-2000	NR (14)	4.25	100	7	Favorable	No	NR	100% surgery + RT	NR
Paulino et al^3^	1976-2001	2 (40)	9.7	NR	NR	NR	No	(0-22 weeks, median 10)	100% RT	No
Whaley et al^5^	1965-2007	9	11.4	66	56	Favorable; better OS for small tumors	No	After-CT	100%RT; 33% surgery + RT	44
Berger et al^9^	1980-2000	10 (26)	4.9	63	52	Favorable	Yes, poor responders (30%)	After-CT	54% surgery; 15% surgery + RT; 35% RT	20
Abdel Rahman et al^10^	1997-2008	20	3	50	67	Favorable only for small tumors	NR	After neo-adjuvant CT	RT (40%), surgery (10%), surgery + RT (25%)	30
Salunke et al^11^	2005-2010	6	1.5	60	NR	NR	No	Surgery at onset	100% surgery at onset, followed by CT and RT	NR
Biswas et al^12^	2023-2011	18 (35)	4.8	68	55	NR; (WBC>11,000/mL and mandible tumors predicted inferior EFS)	NR	After neo-adjuvant CT	RT (72%), surgery (9%)	9
Olson et al^13^	1972-2015	8 (17)	10.4	87	75	NR	No	82% surgery at onset, 16% after neo-adjuvant CT	Post-surgical RT (82%)	12
Torabi et al^2^	2004-2016	143 (284)	5	90	NR	Favorable; (Age<18 years; localized disease)	No	After neo-adjuvant CT	62% RT; 37% surgery; 17% RT + surgery	10
Grevener et al^14^	2009-2019	NR (51)	3.3	87	74	Favorable; Better OS for younger patients, smaller volume and localized disease	No	After neo-adjuvant CT	30% surgery alone; 16% RT; 52% surgery + RT	14
Iatrou et al^15^	2007-2016	6	3.7	60	80	NR	No	After neo-adjuvant CT	66% surgery + RT;	16

Legend: HN ES: head and neck Ewing sarcoma; EFS: event-free survival; OS: overall survival; HD-CT: high-dose chemotherapy; M: metastasis; NR: not reported; WBC: white blood cells; CT: chemotherapy; RT: radiotherapy.

In our series we decided to treat patients with HD-CT even if not strictly indicated in ongoing protocols; in fact, the effective role of HD-CT with autologous stem cell rescue continues to be debated.^16^

One of the major prognostic factors in patients with ES is local disease control. This is particularly challenging for tumors located in the HN, where surgical radicality is limited by the proximity to critical structures and the risk of functional and aesthetic damage: therefore, particular attention should be paid to potential side effects related to both surgical treatment and RT/PT.

Du Bois et al.^17^ supported a subtotal resection followed by RT and CT, with improved outcomes (90.2%) compared to RT alone (58.2% 5-year OS).

Furthermore, maxillofacial surgery in children poses unique challenges due to oncologic, functional, and psychosocial concerns.^6,8,3^ Local flaps and skin grafts are preferred^18^; dermal substitutes may precede grafting. Autologous bone (cranial, rib, iliac crest) is usually used for skeletal reconstruction, while synthetic materials are only temporary.^8^ In our series, all five surgically treated patients underwent reconstruction in the same procedure. Complex flaps like fibular free flaps are reserved for older children; we reported only one failure which occurred in a six-year-old patient, who underwent a second surgery for reconstruction revision, and subsequent setup of a myomucosal flap of the bilateral genian region to reconstruct the integrity of the floor of the mouth. No further surgical complications have been reported. Interestingly, in our series, among the four patients who relapsed, we experienced only one local relapse, a localized ES of the maxillary sinus in a patient who relapsed at 24 months since diagnosis.

Radiotherapy has no clearly defined optimal dose or timing, administered pre- or post-operatively in cases of incomplete resection, typically delivering 45–54 Gy to the tumor bed, with several reports of improved outcomes with dose escalation. Our patients received different doses, ranging from 41.4 to 59.4 Gy, according to ongoing protocol and international guidelines.

Radiotherapy in HN regions is technically difficult and potentially associated with short- and long-term toxicities, including growth abnormalities and secondary malignancies.^19^

In recent years, new techniques have been investigated, such as PT, taking advantage of the typical distribution of proton particles that allow to spare a larger part of normal tissues surrounding tumor target, with decrease rates of acute and late toxicities, including secondary malignancies^9,10^ with a similar outcome compared to RT.^20^ Structured high-quality clinical investigations are needed to evaluate long-term effectiveness.

In our series, one patient with skull ES developed severe toxicity, including brain radionecrosis seven months after RT and corneal ulceration requiring enucleation. Radiation necrosis is a late, often irreversible complication that may cause significant neurological morbidity and mortality. The patient was treated with antioxidants and bevacizumab, achieving partial radiological improvement. Retrospective RT analysis showed that although the cornea was outside the target volume, it received a mean scattered dose of 30 Gy (maximum 45 Gy) in the adjacent region. This is a single case, but it is one of several reported cases which highlighted the need to put more effort and attention into preventing short and long-term sequelae.^19^ No long-term toxicities in our patients treated with PT were reported.

In the literature, OS for HN ES, regardless treatment type, ranges from 53%-100% in different studies.^14^ The median survival in the pooled studies is about 80%. In our series, two-year EFS was 77%, and two-year OS were 88%, consistent with data reported.

In conclusion, HN ES management remained challenging. In most of our cases, we completed intensive chemotherapy and/or RT to maximize response before attempting radical surgery. When feasible, surgery aiming at CR remains a key prognostic factor, particularly in non-metastatic patients, although reconstruction could be complex in growing children. Radiotherapy and PT also carry risks of unpredictable long-term sequelae.

Lastly, metastasis at diagnosis remains the main independent prognostic factor.

Our two-year EFS and OS were 77% and 88%, and five-year OS was 66%, consistent with literature data.

Given the rarity of this localization, a coordinated multidisciplinary team approach with close collaboration among radiation oncologists and maxillofacial surgeons is essential to define standardized treatment strategies, minimize long-term sequelae, and ultimately improve prognosis.
